# Organic photodiode with dual functions of indoor photovoltaic and high-speed photodetector

**DOI:** 10.1007/s12200-022-00024-5

**Published:** 2022-04-28

**Authors:** Tae Wook Kim, Sung Hyun Kim, Jae Won Shim, Do Kyung Hwang

**Affiliations:** 1 Center of Opto-Electronic Materials and Devices, Post-Silicon Semiconductor Institute, Korea Institute of Science and Technology (KIST), Seoul, 02792 Republic of Korea; 2 School of Electrical Engineering, Korea University, Seoul, 02841 Republic of Korea; 3 Division of Nano & Information Technology, KIST School, University of Science and Technology (UST), Seoul, 02792 Republic of Korea

**Keywords:** Organic semiconductor, Photodiode, Indoor photovoltaics, Photodetector

## Abstract

Energy harvesting and light detection are key technologies in various emerging optoelectronic applications. The high absorption capability and bandgap tunability of organic semiconductors make them promising candidates for such applications. Herein, a poly(3-hexylthiophene-2,5-diyl) (P3HT):indene-C60 bisadduct (ICBA) bulk heterojunction-based organic photodiode (OPD) was reported, demonstrating dual functionality as an indoor photovoltaic (PV) and as a high-speed photodetector. This OPD demonstrated decent indoor PV performance with a power conversion efficiency (PCE) of (11.6 ± 0.5)% under a light emitting diode (LED) lamp with a luminance of 1000 lx. As a photodetector, this device exhibited a decent photoresponsivity of 0.15 A/W (green light) with an excellent linear dynamic range (LDR) of over 127 dB within the optical power range of 3.74 × 10^−7^ to 9.6 × 10^−2^ W/cm^2^. Furthermore, fast photoswitching behaviors could be observed with the rising/falling times of 14.5/10.4 μs and a cutoff (3 dB) frequency of 37 kHz. These results might pave the way for further development of organic optoelectronic applications.

**Graphical Abstract:**

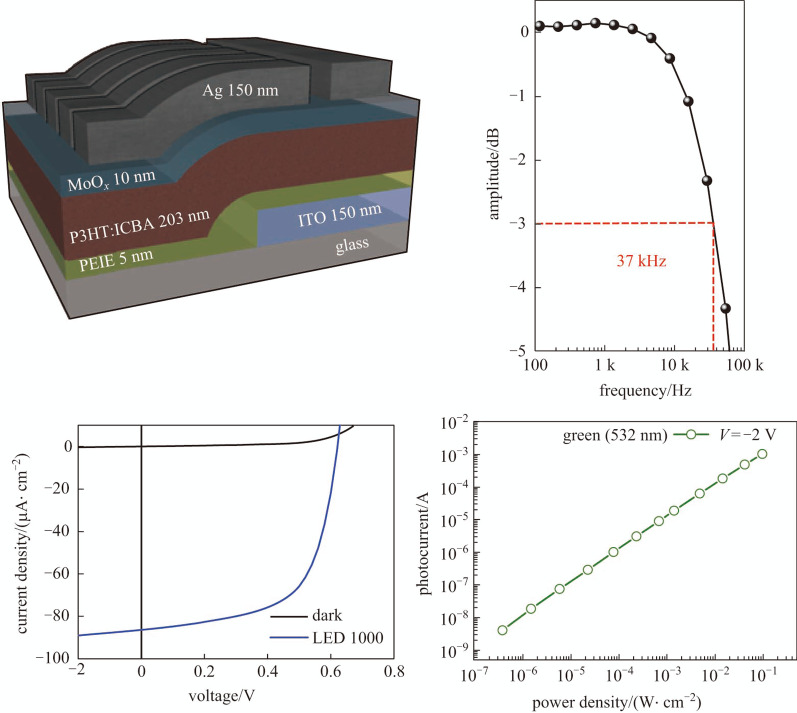

## Introduction

Organic semiconductors are an important class of materials, owing to their great potential in diverse electronic and optoelectronic applications. In particular, indoor energy harvesting and photodetection, have become key technologies for various emerging optoelectronic applications [1], such as low powered or self-powered devices. Organic semiconductors are suitable candidates for these applications because of their distinct features, such as high absorption capability, bandgap tunability, low-cost manufacturing, and compatibility with flexible/wearable devices. Indoor organic photovoltaics (OPVs) have been extensively studied, and considerable efforts have been devoted to improving their power conversion efficiency (PCE). Consequently, PCE values of over 20% have been achieved under fluorescent lamp (FL) or light emitting diode (LED) illuminations [1–4]. In addition, organic photodetectors have attracted considerable research attention due to their high responsivity, wide linear dynamic range (LDR), detection wavelength selectivity, and ease of large area fabrication [5–8]. The two types of photodetectors (PDs) are: (1) photoconductors (or phototransistors) and (2) photodiodes. Phototransistors have a photoconductive gain, resulting in a very high responsivity. However, their photoswitching speed is intrinsically low. In contrast, photodiodes show a lower responsivity, but have a very high response time and an excellent LDR. Furthermore, photodiodes can operate in photovoltaic mode (self-powered operation).

In this paper, we propose a poly(3-hexylthiophene-2,5-diyl) (P3HT):indene-C60 bisadduct (ICBA) bulk heterojunction-based organic photodiode (OPD) exhibiting both indoor PV and high-speed photodetector behaviors. This OPD exhibited decent indoor PV performance with a PCE of (11.6 ± 0.5)% under an LED lamp with a luminance of 1000 lx. As a photodetector, a 400–600 nm spectral photo response was clearly observed, and a relatively high responsivity of 0.15 A/W was achieved under green light illumination. Above all, this diode showed an excellent LDR of over 127 dB within an optical power range of 3.74 × 10^−7^ to 9.6 × 10^−2^ W/cm^2^, and very fast dynamic behavior with the rising/falling times of 14.5/10.4 μs, and a cutoff (3 dB) frequency of 37 kHz.

## Experimental

**Fig. 1:**
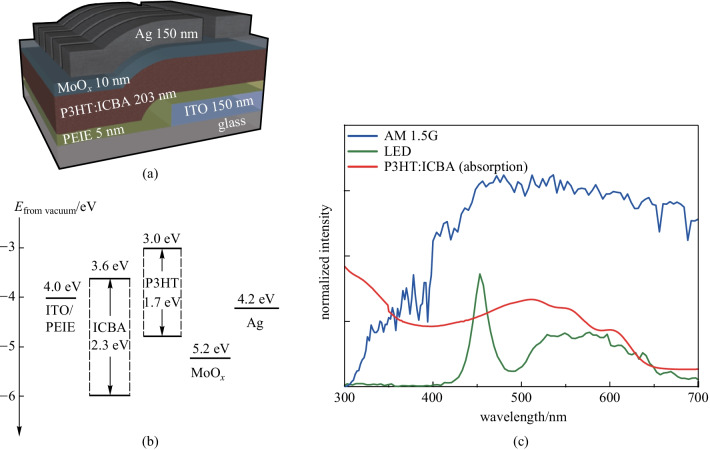
**a** Schematic 3D view of our organic photodiode with an inverted geometry and **b** corresponding energy level diagram. **c** Output spectral irradiances of 1-sun (A.M. 1.5 G) and LED lamp illumination, and absorption spectra of P3HT:ICBA

The OPDs were fabricated on indium tin oxide (ITO)-coated glass substrates. A concentration of 0.1 wt% of ethoxylated polyethyleneimine, PEIE (80% ethoxylated, Mw ∼ 70,000 g/mol, about 37 wt% in water, purchased from Aldrich, St. Louis, Missouri, USA) was diluted with 2-methoxy ethanol (Aldrich, St. Louis, Missouri, USA) and placed overnight on a stirrer at room temperature. The donor P3HT (4002E, Rieke Metals, Lincoln, NE, USA) and acceptor ICBA (Luminescence Technology Corp., Taiwan, China) were mixed in dichlorobenzene (DCB) (Aldrich, St. Louis, MO, USA), where P3HT:ICBA was produced with a total concentration of 40 mg/mL in a 1:1 weight ratio. The blended solution was stirred overnight at 70 °C in an N_2_ gas-filled glove box. For deposition, the ITO substrates (AMG, Republic of Korea) were cleaned with a detergent in an ultrasonic bath for 20 min at 50 °C. Subsequently, the substrates were ultrasonicated in deionized (DI) water, acetone, and isopropyl alcohol sequentially for 20 min at 50 °C, in each case. The PEIE layer (5 nm) was coated via spin coating at a speed of 5000 r/min for 60 s, followed by thermal annealing at 110 °C for 10 min. To deposit the photoactive layer, the samples were loaded into an N_2_ gas-filled glove box. The photoactive layer of the P3HT:ICBA solution was spin-coated at 800 r/min for 30 s and then solvent-annealed overnight. Subsequently, the samples were thermally annealed at 150 °C for 10 min. After loading the samples into a vacuum thermal evaporation system (Daedong High Tech, Republic of Korea) connected to the glove box, a 10 nm thick layer of MoO_3_ and a 150 nm thick layer of Ag were deposited through a shadow mask at a base pressure of 8 × 10^−8^ Torr. Figure 1a and b show the schematic 3D view of our OPDs with an inverted geometry, and the corresponding energy level diagram, respectively.

The current density–voltage (*J–V*) characteristics of the OPV under illumination and dark conditions were measured using a source measurement unit (2401, Keithley Instruments, Cleveland, Ohio, USA). For illumination, two ambient light sources were used: an air mass 1.5 global (G) solar simulator (McScience, Suwon, Republic of Korea) with irradiance, *I*_L_ = 100 mW/cm^2^, and an LED lamp (McScience, Suwon, Republic of Korea) at 1000 lx with an irradiance of 0.23 mW/cm^2^. The output spectral irradiance of the 1-sun illumination and LED lamp illumination are shown in Fig. 1c. The OPVs’ external quantum efficiency (EQE) was gauged via an incident photon-to-current efficiency measurement system (McScience, Suwon, Republic of Korea). Using an optical microscope, the active area of each cell was determined to be approximately 0.1 cm^2^. The thickness of each layer was measured using an alpha-step (Alpha-Step IQ, KLA-Tencor, California, USA). Two light sources were used for photodetection characterization: a tungsten–halogen lamp (200 W) with a monochromator (Princeton Instruments, SP2150) for recording spectral photo responses, and a 532 nm laser diode (LD, Thorlabs, DJ532-40) for examining the dependence of performance parameters as a function of the illumination intensity. All static electrical and photo response measurements were performed using a semiconductor parameter analyzer (Agilent 4156C) in the dark and under illumination. The dynamic on/off photoswitching behaviors were examined using a function generator (Tektronix AFG31000) to operate the 532 nm LDs. The photoelectrical bandwidth measurement system consisted of a function generator (Tektronix AFG31000), 532 nm LD, an SR-570 low-noise-current preamplifier, and an SR-830 lock-in amplifier.

## Results and discussion

**Fig. 2:**
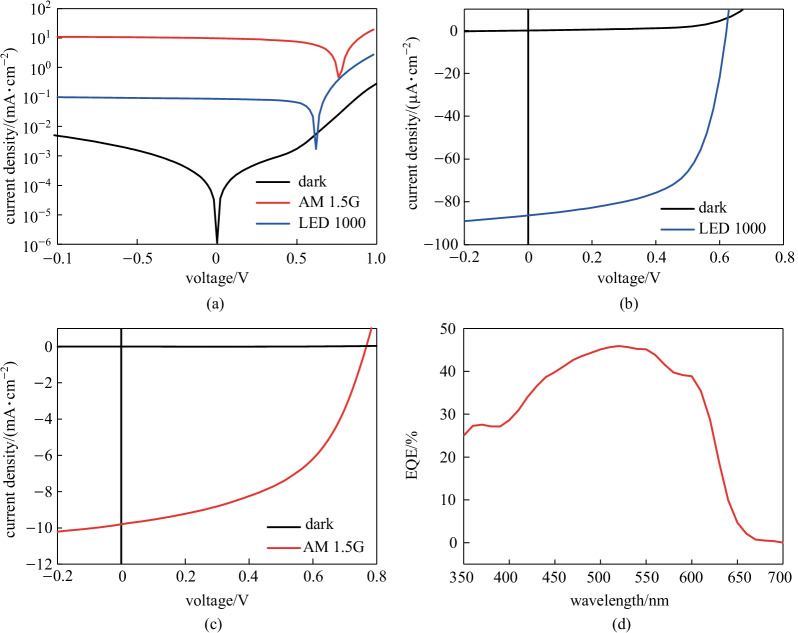
OPV performance. Representative current density–voltage characteristics on **a** the logarithmic scale, and **b** and **c** the linear scale under LED lamp illumination (1000 lx) and 1-sun illumination. **d** External quantum efficiency (EQE) spectra under 1-sun illumination

Figures 2a–c show the representative current density–voltage characteristics on the logarithmic and linear scales under LED lamp illumination (1000 lx) and 1-sun illumination. Under AM 1.5 G illumination, the devices exhibited an open circuit voltage (*V*_OC_) of (0.766 ± 0.004) V, a short circuit current (*J*_SC_) of (9.1 ± 0.7) mA/cm^2^, fill factor (FF) of (50.95 ± 0.76)%, and resultant PCE of (3.6 ± 0.3)% (averaged over 5 devices). In contrast, *V*_OC_ = (0.65 ± 0.029) V, *J*_SC_ = (81.7 ± 4.5) μA/cm^2^, FF = (61.34 ± 1.92)%, and PCE = (11.6 ± 0.5)% were observed under an LED lamp with a luminance of 1000 lx (indoor condition). The PV performance parameters are listed in Table 1. The external quantum efficiency (EQE) spectra are directly correlated with *J*_SC_, which is mainly affected by the absorption spectra. As expected, the EQE spectra of our devices were in good agreement with the absorption spectra (Fig. 2d and 1c). The values of the photovoltaic performance parameters obtained demonstrate that the P3HT:ICBA bulk heterojunction system was able to function as an indoor PV [9–11] (Table 1).

**Table 1:** Photovoltaic performance parameters (averaged over five devices) and comparison with previous work

Light source	Material	*V*_OC_/mV	*J*_SC_/(AM 1.5: mA·cm^−2^) (LED: μA·cm^−2^)	FF/%	PCE/%	Reference
AM 1.5 G	P3HT:ICBA	766 ± 4	9.1 ± 0.7	50.95 ± 0.76	3.6 ± 0.3	This work
LED	P3HT:ICBA	650 ± 29	81.7 ± 4.5	61.34 ± 1.92	11.6 ± 0.5	This work
LED	P3HT:ICBA	730 ± 10	50 ± 1.0	63 ± 1	13.05 ± 0.42	[9]
LED	P3HT:ICBA	698 ± 4	37.4 ± 0.9	68.2 ± 3.5	10.4 ± 0.4	[10]
LED	P3HT:ICBA	713 ± 3	68.2 ± 1.6	74.9 ± 0.4	13.0 ± 0.3	[11]

**Fig. 3:**
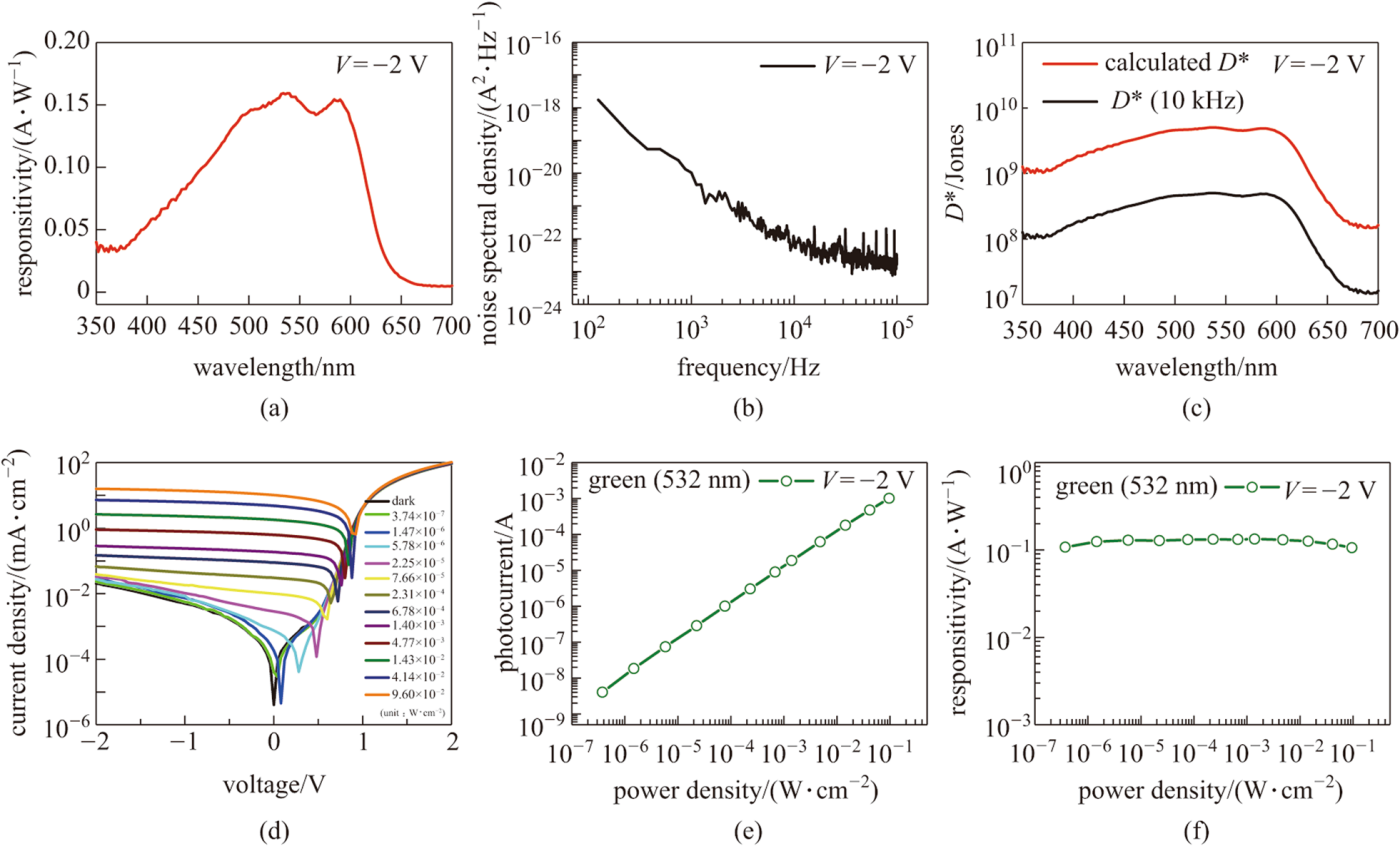
Photodiode static performance. **a** Spectral responsivity characteristics, **b** noise power spectral densities, and **c** specific detectivity as a function of illumination wavelength. **d** Dark and photoinduced current density–voltage characteristics under different green LD (532 nm) illumination power densities. **e** and **f** Variations of photocurrent and responsivity as functions of incident light power density

Subsequently, the photodetection characteristics were investigated. Figure 3a shows the spectral responsivity (*R*) under monochromatic illumination from a tungsten–halogen lamp at an applied voltage of –2 V (photoconductive mode). Maximum responsivity values of 0.15 A/W were obtained under 500–600 nm light illumination. The spectral responsivity was also in agreement with the previous EQE and absorption spectra. The specific detectivity (D*) is an important figure of merit for evaluating photodetectors; it is expressed as $D^{*} = {{R\left( {A\Delta f} \right)^{1/2} } \mathord{\left/ {\vphantom {{R\left( {A\Delta F} \right)^{1/2} } {\left( {\left\langle {I_{n}^{2} } \right\rangle } \right)}}} \right. \kern-\nulldelimiterspace} {\left( {\left\langle {I_{n}^{2} } \right\rangle } \right)}}^{1/2}$ [12], where *R* is the responsivity, *A* is the active area of the device, Δ*f* is the spectral bandwidth (set to 1 in the measurement system for this work), and $\left( {\left\langle {I_{n}^{2} } \right\rangle } \right)^{1/2}$ is the root-mean-square dark noise current obtained from the noise power spectral densities (Fig. 3b) measured using an SR-570 low-noise-current preamplifier and an Advantest R9211B digital spectrum analyzer. Taking the measured spectral responsivity and device area (0.1 cm^2^) into account, the D* values were estimated to be 10^8^–10^9^ Jones in the spectral range of 350–650 nm, as shown in Fig. 3c. The theoretical D* values dominated by the shot noise limit can be calculated using the equation D* = *R*(*A*)^1/2^/(*2qI*_dark_)^1/2^ [13], where *I*_dark_ denotes the dark current and *q* is the elementary charge; which, in our case, generated D* values in the range 10^9^–10^10^ Jones. Both the measured and theoretical D* values were two or three orders of magnitude lower than those of the state-of-the-art OPDs or commercial Si PDs (10^12^ Jones). This might have been caused by a higher dark leakage current density of the order of 10^−3^ mA/cm^2^. Further optimization, such as increasing the thickness of the P3HT:ICBA layer, can enhance D* by reducing the dark leakage current density.

Another important characteristic of photodetectors is the dependence of their performance parameters on the illumination power. To examine this, a green LD (532 nm) was selected owing to its higher photocurrent. Figure 3d shows the dark and photoinduced current density–voltage characteristics at different incident optical power densities (from 3.74 × 10^−7^ to 9.6 × 10^−2^ W/cm^2^). As the illumination power density increased, the photocurrent linearly increased. It is worth noting that the photocurrent was not saturated within the measured range, as shown in Fig. 3e. This indicates that the photogenerated carriers in our OPD were readily collected without serious degradation. The LDR is directly correlated with the photocurrent linearity as a function of the illumination power and can be expressed as LDR = 20log(I*_ph_/*I*_dark_) [9, 14], where *I*_dark_ denotes the dark current, and I*_ph_ denotes the photocurrent measured at the highest optical power. The LDR of our OPD was calculated to be 127 dB, which agrees with that of a commercial photodetector (Si or InGaAs) [14]. Such outstanding linearity characteristics resulted in negligible variations in responsivity regardless of the illumination power, as shown in Fig. 3f.

**Fig. 4:**
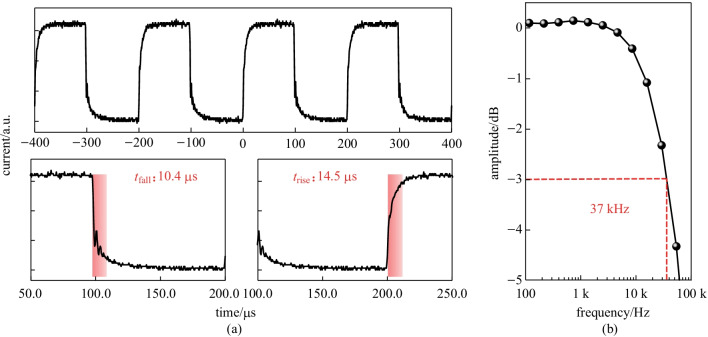
Dynamic behaviors. **a** Temporal photocurrent changes at green LD (532 nm) modulation frequency of 5 kHz; the rising and falling times are 14.5 and 10.4 μs, respectively. **b** Normalized photoresponse as a function of frequency

In addition to the static characteristics, the dynamic behaviors of our OPDs in response to a light pulse are also very important from the perspective of photodetectors. Figure 4a shows the temporal photocurrent characteristics under green (532 nm) LD light pulse illumination at a modulation frequency of 5 kHz. There were no significant reductions or delays in the photoswitching behaviors. The rising and falling times were estimated to be 14.5 and 10.4 μs, respectively. To investigate the cutoff (3 dB) frequency of our OPDs, we used a photoelectrical bandwidth system consisting of a light source (green LD), function generator, low-noise-current preamplifier, and lock-in amplifier. Figure 4b shows the normalized amplitude (photo response) as a function of the green LD light pulse frequency. The 3 dB frequency was measured to be 37 kHz. Such a fast response is comparable to that of a state-of-the-art P3HT:ICBA-based OPD [6]. For comparison, the performance parameters of our device and other OPDs previously reported in the literature are summarized in Table 2.

**Table 2:** Comparison of figures of merit for organic photodetectors

Material	*R*/(A·W^−1^)	D*/Jones	LDR/dB	Response time (3 dB frequency)	Reference
P3HT:ICBA	0.15	10^9^–10^10^	127	14.5 μs, 10.4 μs (37 kHz)	This work
P3HT:ICBA	0.25	10^13^	160	35 μs (15 kHz)	[6]
P3HT:PCBM		10^12^	89		[8]
PDDTT:PCBM	0.17	10^13^	100		[5]

## Conclusion

We demonstrated P3HT: ICBA-based OPDs that functioned both as indoor photovoltaics and high-performance photodetectors. Our OPDs demonstrated reasonable indoor PV performance with a PCE of (11.6 ± 0.5)% under an LED lamp with a luminance of 1000 lx, and preferable photodetection properties with 400–600 nm spectral photo response with a relatively high responsivity of 0.15 A/W. This device demonstrated an excellent LDR of over 127 dB and a rapid dynamic response with a cutoff frequency of 37 kHz. Hence, we believe that our results will lay the foundation for further development of organic optoelectronic devices.
